# Yishentongluo decoction in treatment of idiopathic asthenozoospermia infertility: Study protocol for a randomized controlled trial

**DOI:** 10.1097/MD.0000000000022662

**Published:** 2020-10-23

**Authors:** Qi Zhang, Lipeng Fan, Fangyuan Li, Zixue Sun, Chenming Zhang, Rubing Chen

**Affiliations:** a College of Clinical Medicine, Chengdu University of Traditional Chinese Medicine, NO.37 Shi-er-qiao Road, Chengdu 610075, Sichuan Province, P.R. China; Chengdu University of Traditional Chinese Medicine Affiliated Hospital, NO.39 Shi-er-qiao Road, Chengdu 610075, Sichuan Province; b Department of Reproductive Medicine, Henan Province Hospital of Traditional Chinese Medicine, NO.6 Dong-feng Road; c Second Clinical Medical School, Henan University of Chinese Medicine, NO.156 Jin-shui-dong Road; d Department of Andrology, The First Affiliated Hospital of Henan University of Chinese Medicine, NO.19 Ren-min Road, Zhengzhou, Henan Province, P.R. China.

**Keywords:** idiopathic asthenozoospermia, male infertility, randomized controlled trial, traditional chinese medicine, yishentongluo decoction

## Abstract

**Background:**

The reproductive dilemma faced by men has always been the focus of the whole society. Idiopathic asthenozoospermia (AZS), as one of the common causes of male infertility, lack of specific treatment. Traditional Chinese medicine has shown potential benefits in the management of male infertility. Yishentongluo decoction (YSTL) is a representative Chinese herbal formula; however, there is still no rigorous clinical trial supporting its application. Therefore, we designed a randomized controlled trial to evaluate the efficacy and safety of YSTL for patients with idiopathic AZS and explain the possible action mechanisms of YSTL in improving sperm motility.

**Methods:**

In this randomized controlled study, a total of 160 eligible patients will be assigned to YSTL group or the Levocarnitine oral solution group in a 1:1 ratio. The treatment period will be 12 weeks and the follow-up period will last 4 weeks. The primary outcome will be the the progressive (motility), sperm rate (%). Secondary outcomes will include the progressive (motility) + non-progressive (motility) sperm rate(%), total effective sperm count, inner mitochondrial membrane potential (MMP) in spermatozoa, and spouse pregnancy rate (%). Safety outcomes will cover electrocardiogram , blood tests (including blood routine test, hepatic function, and renal function), urine routine test, and stool routine test. The semen parameters, sperm MMP test, and all the safety outcomes will be performed at the baseline, 4th, 8th and 12th week. The pregnancy outcome will be evaluated at 4 weeks after treatment.

**Discussion:**

This study will provide initial evidence regarding the efficacy and safety of YSTL in the treatment of idiopathic AZS with kidney deficiency and blood stasis pattern. In addition, potential mechanisms of YSTL in improving sperm motility will be explored based on sperm MMP test.

**Trial registration:**

Chinese Clinical Trials Register identifier, ChiCTR2000033290, registered on 26 May 2020.

## 1 Introduction

Infertility is defined by the World Health Organization (WHO) as “the inability of a sexually active, non-contracepting couple to achieve pregnancy after 12months,”^[1]^ and it affects around 10% to 15% of couples all over the world.^[2]^ Male infertility, which is mainly associated with disorders in sperm such as low sperm count, poor sperm quality, or both, accounts for up to 50% of all infertility cases.^[3]^ Among them, decreased motility (asthenozoospermia, AZS) which is characterized by reduced spermatozoa forward motility (progressive motility < 32%),^[4]^ is one of the most frequent types of male infertility, and affects approximately 40% of all cases.^[5]^ Idiopathic asthenozoospermia was defined when the etiology was unknown. For decades, despite greater understanding of male infertility in recent years, idiopathic sperm abnormalities still account for about 30 percent of male infertility.^[6]^ Male infertility has a seriously negative impact on a man's physical, psychological, emotional well-being, especially family harmony, or even social relationships.^[7]^ Being a destructive and complicated disorder, male infertility attracting more and more public attention.

Male reproduction is a complex process, the most critical factor for male fertility is sperm motility, and any failures in sperm production, maturity, storage, or expulsion will lead to infertility finally.^[8]^ Asthenozoospermia can be a consequence of genital tract infection, abnormal hormone levels, varicocele, systemic diseases, environmental factors, or be the result of changes that have not yet been recognized at the molecular level.^[9–15]^ Recently, mitochondrial dysfunction reported in AZS men came to attention, several studies reported this dysfunction is related to idiopathic asthenozoospermia, since sperm motility is regulated by intrinsic factors, and requires an appropriate supply of adenosine triphosphate.^[16–18]^ For adenosine triphosphate synthesis, these gradients are generated by electron transfer maintained by the inner mitochondrial layer. Then energy storage in mitochondria is related to proton concentration and electric potential gradient in cell membrane. The energy state of mitochondria is described by mitochondrial membrane potential (MMP), which regulates intact functional mitochondria and is significantly correlated with the motility of sperm.^[19]^ Thus, we said the functional competence of the sperm can be evaluated by measuring the inner MMP in spermatozoa.^[20]^ It has been observed that highly progressive motility sperms do have higher MMP as compared to infertile males with impaired motility, and they are usually morphologically normal and can undergo acrosome reaction invariably at the same time.^[21,22]^ Therefore, analyse the function of mitochondria may provide a way to distinguish sperm motility and MMP can be considered as a potential regulator and indicator of sperm motility.^[8]^

Due to unknown etiology and complex pathogenesis, there are no specific therapies to effectively treat the idiopathic asthenozoospermia infertility, and a number of drugs are empirically managed using in the clinic.^[23]^ Some studies of antioxidants have tended to show that semen parameters may benefit from supplements of carnitine,^[24]^ vitamin E,^[25]^ Selenium,^[26]^ or N-acetylcysteine.^[27]^ However, the therapeutic potential of antioxidant remains debated, since limited by sample size.^[28]^ In recent years, L-Carnitine (LC) has attracted extensive attention in treating male infertility. LC which is mainly of exogenous origin not synthesized is an essential substance in the process of fatty acid oxidation, and plays an important role in the production and transport of energy in cells.^[29]^ In the study of Lenzi and collaborators, an interesting aspect has been found: there is a high concentration of carnitine in the male reproductive tract, particularly in the epididymis, representing its key role in energy metabolism and sperm maturation.^[30]^ Given this finding, it is now known that the accumulation of LC in ejaculation may be a marker of epididymal function.^[28]^ LC has been used as a treatment approach in patients with idiopathic asthenozoospermia, just mainly based on the crucial role of it in energetic metabolism and its concentration in epididymis.^[31]^ Some controlled and uncontrolled studies suggest the potentially positive effects of LC and its acyl derivatives in the treatment of some forms of oligoasthenoteratozoospermia.^[32,33]^ Then, recent clinical studies confirmed LC has been successful in treating men with asthenospermia.^[34]^

However, some treatments including LC are sometimes inefficient, costly, or related to side effects, about half of patients want to seek more alternative therapies such as Traditional Chinese medicine (TCM) to cure AZS.^[35,36]^ TCM has been used for hundreds of years in clinical practice to treat male infertility.^[37]^ In the theory of TCM, the kidney is the place to store kidney essence, which plays an important role in reproduction. Most experts believe that idiopathic AZS is closely associated with the dysfunction of the viscera, especially the disorder of the kidney.^[38]^ Several studies have revealed the active components of herbal medicine that nourish kidney-essence, support blood circulation in the reproductive system, and control testosterone secretion.^[39]^ They have been shown to significantly improve sperm motility in animal studies.^[40]^ And also shown positive effects in the enhancement of the parameters of sperm in men with infertility.^[13,41]^ Some small-sample clinical trials have confirmed the efficacy of Yishentongluo decoction (YSTL) as complementary therapy for idiopathic asthenospermia.^[42]^ Our previous study suggested that YSTL significantly improved sperm quality and sperm deoxyribonucleic acid integrity in patients with varicocele-associated asthenospermia combined with minimally invasive surgery.^[43]^ However, the present clinical study has the limitations of the methodology and sample size, and sufficient data on YSTL as complementary therapy compared with western drugs in idiopathic AZS are lacking. Thus, we aimed to design an randomized controlled trial to evaluate the efficacy and safety of YSTL in idiopathic AZS, and to explore the possible mechanism of it.

## 2 Methods and analysis

### 2.1 Design

**Figure 1:**
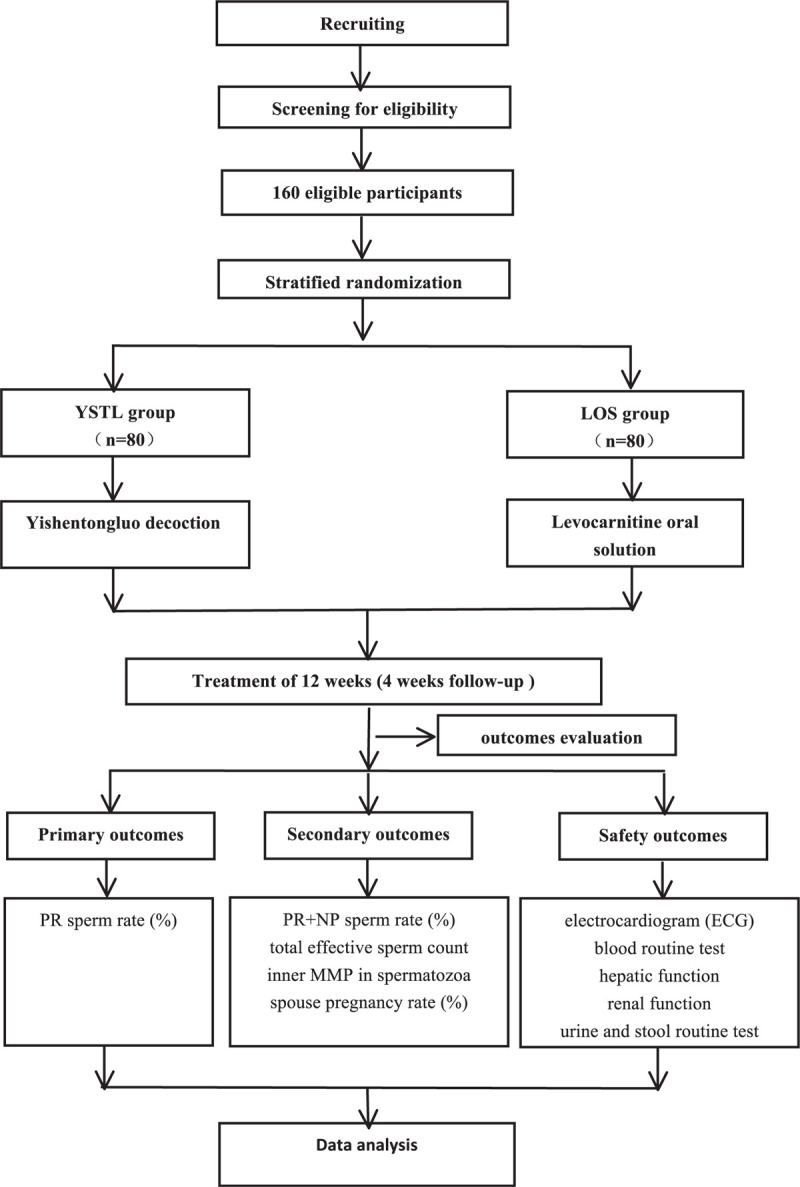
Trial flow chart.

A 12-weeks, single-center, assessor-blinded, randomized controlled trial is currently being performed. YSTL will be compared with Levocarnitine Oral Solution to evaluate the efficacy and safety of YSTL for patients with idiopathic AZS. The trial will be conducted in the Department of reproductive medicine, Henan Province Hospital of TCM. All participants will be required to provide written informed consent prior to entering the trial. The study schedule is detailed in Table 1. The flowchart of the trial is shown in Figure 1. The Standard Protocol Items: Recommendations for Interventional Trials checklist is provided as Additional file 1.

**Table 1:**
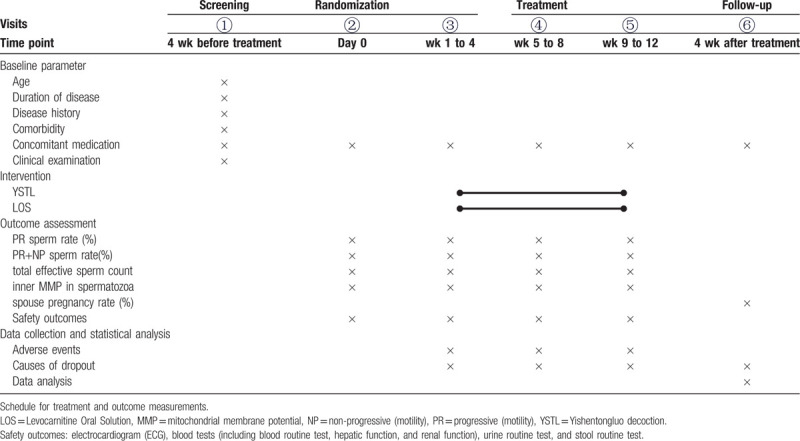
Data collection points.

### 2.2 Ethic approval

The research protocol is performed in accordance with the principles of the Declaration of Helsinkii^[44]^ and has been approved by The Ethics Review Board of Henan Province Hospital of TCM (approval no. 2020-1120-2) and followed up the whole process. We registered the study on the Chinese Clinical Trial Registry (Registration No. ChiCTR2000033290).

### 2.3 Participants

A total of 160 male patients with idiopathic AZS infertility will be enrolled in this study after their informed consents are obtained. We will post the printed recruitment posters in the hospital or release recruitment information on the hospital social media (QQ, WeChat, Weibo) to recruit participants. All participants should meet the diagnostic criteria of idiopathic AZS infertility and TCM pattern differentiation criteria of Kidney deficiency and Blood stasis. If they meet the study criteria, they will be invited to the Department of reproductive medicine, Henan Province Hospital of TCM to undergo the study. Eligible participants will be randomized at a 1:1 ratio to the YSTL group or the Levocarnitine Oral Solution (LOS) group. The course of treatment will last 12 weeks. The pregnancy of the wife will be followed up at 4 weeks after treatment.

#### 2.3.1 Diagnostic criteria

Diagnostic criteria of idiopathic AZS infertility will be based on the WHO laboratory manual for the examination and processing of human semen, 5th ed, in 2010.^[4]^ To be specific, the following five criteria should all be met:

(1) the inability of a sexually active, non-contracepting couple to achieve pregnancy after 12months, due to the reason of the man;
(2) two or more semen examinations (abstinence time for 3–7 days each time) of male suggested asthenozoospermia, AZS: progressive (motility) (PR) < 32%, or PR + non-progressive (motility) (NP) < 40%;
(3) sperm concentration > 15 × 10^6^/mL;
(4) proportion of normal sperm ≥ 4%; and
(5) no obvious causative factors were found.

The TCM pattern differentiation criteria of Kidney deficiency and Blood stasis will refer to the Guiding Principles of New Herbs Research^[45]^ and the Guidelines for Diagnosis and Treatment of Male Infertility with Integrated Traditional Chinese and Western Medicine (Trial Edition).^[46]^ The standards of Kidney deficiency and Blood stasis were separated into major symptoms and secondary symptoms. Major symptoms: Couples of childbearing age who have lived together for more than one year, have regular sexual life, not taken any contraceptive measures, and the woman cannot become pregnant due to the man's reasons. Secondary symptoms:

(1) soreness and weakness of waist and knees;
(2) dizziness and tinnitus;
(3) dark tongue body with ecchymosis or petechia;
(4) thick and tortuous sublingual veins; and
(5) a deep and weak pulse, or a deep and unsmooth pulse.

Patients were considered to have Kidney deficiency and Blood stasis if they met all of the major symptoms and two secondary symptoms such as a and c, or a and d, or b and c, or b and d, and met the pulse manifestation.

#### 2.3.2 Inclusion criteria

Participants who meet all of the following conditions will be included. The inclusion criteria are as follows:

(1) males, aged between 23 and 48 years;
(2) confirmed the diagnosis of male infertility and idiopathic AZS;
(3) confirmed the TCM pattern differentiation criteria of Kidney deficiency and Blood stasis;
(4) with normal sexual function, regular sexual life (the frequency of sexual life is required to be no less than 1 time/week); and
(5) willing to join this research and sign an informed consent form.

#### 2.3.3 Exclusion criteria

The exclusion criteria are as follows:

(1) with infertility caused by organic lesions of the reproductive system or female infertility;
(2) with mixed antiglobulin reaction test for antisperm antibodies (+);
(3) with abnormal sex hormone, abnormal seminal plasma biochemistry, or abnormal seminal plasma elastase;
(4) with infertility caused by the unable to complete sexual intercourse, including but not limited to erectile dysfunction, ejaculatory disorder;
(5) with reproductive system infection, such as chlamydia trachomatis or mycoplasma infection;
(6) with a history of allergy to treatment drugs or allergic constitution;
(7) use drugs affecting the experimental study within 3 months;
(8) complicated with mental diseases, malignant tumor, or serious organic diseases;
(9) participated in other clinical trials in the past 3 months.

### 2.4 Sample size

Sample size was estimated based on the primary study results. Our previous pre-experiment have shown that the effective rate (*P*_1_) of the YSTL group (treatment group) is 79%, and the effective rate (*P*_2_) of the LOS group (control group) is 54%. The the following formula was used to estimate sample size: 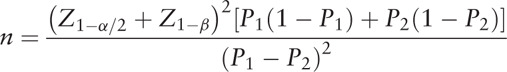


with a 5% significance level (*α*= 0.05, two-sided) and 90% power (*β*= 0.1), the final sample size has been set at a total of 160 patients (80 in each group), assuming a 15% dropout rate.

### 2.5 Randomization and Blinding

Eligible participants who provide informed consent will be randomized at a 1:1 ratio into either YSTL group receiving a 12-week YSTL therapy or LOS group receiving the Levocarnitine Oral Solution therapy. Randomization will be performed based on a random list of numbers generated by SPSS21.0 software (International Business Machines Corp., Armonk, NY). An independent researcher will prepare the assignments in opaque envelopes containing an allocation sequence number and be responsible for the concealment of allocation sequence. Due to the specific nature of the intervention, it is not possible to blind the participants and personnel involved in conducting the trial. Outcome assessors, data managers, and statisticians will be unaware of the treatment allocations.

## 3 Interventions

### 3.1 Basic interventions

Basic interventions will be given to the two groups including improvement of lifestyle to eliminate negative factors for physical and psychological, such as relief of tension, reduction of extra fat, avoid smoking or alcohol, appropriate of exercise, maintaining a comfortable mood.

### 3.2 Drug intervention

#### 3.2.1 YSTL group

YSTL consists of 12 g of Cuscuta chinensis Lam, 12 g of Epimedium brevicornu Maxim, 10 g of Rehmannia glutinosa, 12 g of Astragalus propinquus Schischkin,12 g of Salvia miltiorrhiza Bunge, and 10 g of Cyathula officinalis Kuan. Participants will be required to take the YSTL granules 2 times a day, 1 grid once a time, 0.5 hours after breakfast and dinner for 12 weeks. The granules produced by Sichuan Neo-green Pharmaceutical Technology Development Co., Ltd. (Sichuan, China) will be used in this study. The above-mentioned crude herbs will be extracted into granules, and the production process will meet the standard of Good Manufacturing Practice.

#### 3.2.2 LOS group

Participants in this group will be required to take the Levocarnitine Oral Solution, 10 mL/piece, 3 times a day, 10 mL once a time, oral after meals. Levocarnitine Oral Solution will be manufactured by Northeast Pharmaceutical Group Shenyang No.1 Pharmaceutical Co., LTD. 12 weeks is one course of treatment and the participants will receive one course of medication. All drugs will be used should be the same lot number.

### 3.3 Combined treatment regulations

During the study, it is forbidden to add other Chinese and western drugs or interventions (such as acupuncture, cupping, and massage) that may interfere with the trial. For participants with other existing conditions, if combined treatment cannot stop, the accompanying intervention should be recorded name, dosage, and the number of times in detail. If the disease progresses during the study, participants can withdraw from the study and use other treatment methods. The case will be treated as an excluded case, and patients will be required to complete the relevant examinations and assessments as much as possible.

## 4 Outcome measures

### 4.1 Primary outcome

The primary outcome in this trial is PR sperm rate (%) at baseline and the 4th, 8th, and 12th week after randomization.

### 4.2 Secondary outcomes

The secondary outcomes will include:

(1) semen parameters including PR+NP sperm rate (%), and total effective sperm count;
(2) the inner MMP in spermatozoa; and
(3) spouse pregnancy rate (%).

The semen parameters and sperm MMP test will be performed at the baseline, 4th, 8th and 12th week after randomization. The pregnancy outcome will be evaluated at 4 weeks after treatment.

### 4.3 Safety outcomes

Safety outcomes will include electrocardiogram , blood tests (including blood routine test, hepatic function, and renal function), urine routine test, and stool routine test. All the safety outcomes will be evaluated at baseline, 4th, 8th, and 12th week. YSTL is an herbal formula that has been used clinically for many years and has satisfactory safety profiles. A general physical examination will be provided at every visit. If an adverse event occurs, clinical investigators will record it in a case record form in detail (including symptoms, occurrence time, duration, examinations, and outcomes). Serious adverse reactions will be reported to the ethical committee of Henan Province Hospital of TCM and rescue procedures will be initiated immediately.

### 4.4 Quality control and trial monitoring

Before the start of the trial, all the researchers will receive special training to guarantee the quality of the study. The training includes how to select and exclude participants, how to complete randomization, how to implement interventions correctly, how to record case report forms in a standard way, how to assess outcomes and manage data. Clinical researchers responsible for diagnosis and treatment will be registered TCM practitioners. To improve the compliance of participants, investigators will provide health education, and fully respect the informed consent right of them. Raw data will be recorded in the case record form, and 2 data managers will input the data into the spreadsheet and recheck the data respectively. To ensure the objectivity of the data, the assessment and statistics will be blinded during the trial. The principle researcher will supervise the whole trial.

### 4.5 Statistical analysis

Statistical Product and Service Solutions (SPSS21.0, International Business Machines Corp., Armonk, NY) software will be performed using for statistical analysis of data by professionals who will be blinded to the whole trial process. Analysis of the efficacy and safety will be based on the principle of intention-to-treat that all random participants will be analyzed. The missing value will be handled by the method of multiple imputations. Data will be presented as mean with standard deviation, median with range, or number with percentage, and different data types will be analyzed by appropriate methods. A repeated-measures multifactorial analysis will be used to analyze value changes of PR sperm rate, PR+NP sperm rate, total effective sperm count, and inner MMP in spermatozoa (See Table 1). The rest of the secondary outcomes will compare the different spouse pregnancy outcomes. Safety outcomes will be compared with the incidence of adverse events in two groups using the *χ*^2^ test. All the statistical tests will be conducted by bilateral difference tests, and if a *P* value is less than 0.05, the data difference will be considered statistically significant.

## 5 Discussion

Increasing male fertility problems have been reported all over the world, while idiopathic Asthenozoospermia (AZS) is a major adverse factor resulting in male infertility, which is diagnosed by reduced sperm motility, progressive motility < 32%, absent of causes (WHO, 2010).^[4,6]^ Since lack of a cause for the worsened semen quality, idiopathic AZS is the most often and most troublesome one among the types of male infertility.^[47]^ AZS can not just be considered as a simple condition in isolated diseases, on the contrary, it is always associated with a comprehensive symptom.^[48]^ Hence, patients with AZS represent a very diverse group actually, and treating this particular patient group is not an easy task. Some scholars found that food supplements may improve seminal fluid conditions by providing energy to male germ cells and protecting these cells from oxidative stress.^[49]^ However, many drugs be used to treating idiopathic male infertility is empirically managed, and the data of drug efficacy are highly heterogeneous.^[23]^ There is no specific treatment modality to treat idiopathic reducing of spermatozoa forward motility by modern medicine, therefore, seeking complementary and alternative therapy such as traditional Chinese herbs is of great significance for the treatment and fertility protection in the infertile men.

Traditional Chinese Medicine (TCM) has been used in clinical practice on male infertility for hundreds of years. Several research data illustrate that the pathogenesis of male infertility is mainly related to dysfunctional mechanisms in the kidney, liver, and spleen, with the involvement of phlegm and blood stasis.^[13]^ The goal of therapy is to balance the reproductive energy (qi), blood, and the yin and yang of internal organs. Treatment based on syndrome differentiation is the most representative character of TCM in recognizing and treating diseases.^[50]^ Therefore, we regard syndrome differentiation as one of the inclusion criteria of participants, namely kidney deficiency and blood stasis. YSTL has been proved as an effective prescription for treating male infertility diagnosed for kidney deficiency and blood stasis. Some studies have reported that the TCM components of Cuscuta chinensis Lam., Epimedium brevicornum Maxim., and Rehmannia glutinosa can reduce reactive oxygen species, improve sperm motility, and effectively increase pregnancy rate.^[51]^ The other active ingredients of herbal medicine in YSTL can also give a highly balanced supply of minerals, antioxidants and nutrients.^[43–44]^

Currently, there is a lack of high-quality research and evidence on Chinese medicine for idiopathic Asthenozoospermia. Therefore, we are conducting a randomized controlled study to evaluate the efficacy and safety of YSTL for patients with idiopathic AZS. This study is a positive-drug parallel randomized controlled trial, we choose LC as the positive-drug since the current evidence indicates that LC can improve spontaneous pregnancy and semen parameters in the treatment of idiopathic oligoasthenozoospermia, with no serious adverse reactions.^[52]^ According to existing studies, sperm motility depends on mitochondrial function and could be better evaluated by the measurement of mitochondrial membrane potential (MMP).^[53]^ We use the PR sperm rate (%) as the main efficacy indicator of this trial. The PR+NP sperm rate (%), total effective sperm count, spouse pregnancy rate (%), and inner MMP in spermatozoa are the secondary efficacy indicators, to provide more reference for efficacy evaluation. Besides, electrocardiogram, blood tests (including blood routine test, hepatic function, and renal function), urine routine test, and stool routine test are safety indicators. The treatment duration will be 12 weeks, include 4-week follow-ups after the treatment, providing reliable results for prognosis assessment and adverse reaction observation.

A limitation in this trial is that blinding is impossible due to the nature of the intervention. Every effort will be made to ensure that the outcome assessors, data managers, and data analysts are blinded to the treatment allocations. Strictly follow the inclusion and exclusion criteria to improve the homogeneity of the subjects. We hope the results will provide preliminary evidence for the efficacy and safety of YSTL in the treatment of idiopathic AZS with kidney deficiency and blood stasis pattern. The detection of inner MMP in spermatozoa may explain the possible action mechanisms of YSTL in improving sperm motility.

## Acknowledgments

We would like to thank all the patients who will participate in the trial and the staff for their support.

## Author contributions

Qi Zhang and Lipeng Fan contributed equally to this study.

**Conceptualization:** Qi Zhang, Lipeng Fan, Zixue Sun.

**Investigation**: Fangyuan Li.

**Supervision:** Chenming Zhang, Rubing Chen.

**Writing – original draft**: Qi Zhang, Lipeng Fan.

**Writing – review & editing**: Qi Zhang, Zixue Sun.
